# Dependence of Piezoelectric Discs Electrical Impedance on Mechanical Loading Condition

**DOI:** 10.3390/s22051710

**Published:** 2022-02-22

**Authors:** Niharika Gogoi, Jie Chen, Jens Kirchner, Georg Fischer

**Affiliations:** Institute for Electronics Engineering, Friedrich-Alexander-Universität Erlangen-Nürnberg (FAU), 91058 Erlangen, Germany; jie_chen97@hotmail.com (J.C.); jens.kirchner@fau.de (J.K.); georg.fischer@fau.de (G.F.)

**Keywords:** piezoelectric, impedance, electrical model, wide-range frequency

## Abstract

The piezoelectric effect, along with its associated materials, fascinated researchers in all areas of basic sciences and engineering due to its interesting properties and promising potentials. Sensing, actuation, and energy harvesting are major implementations of piezoelectric structures in structural health monitoring, wearable devices, and self-powered systems, to name only a few. The electrical or mechanical impedance of its structure plays an important role in deriving its equivalent model, which in turn helps to predict its behavior for any system-level application, such as with respect to the rectifiers containing diodes and switches, which represent a nonlinear electrical load. In this paper, we study the electrical impedance response of different sizes of commercial piezoelectric discs for a wide range of frequencies (without and with mechanical load for 0.1–1000 kHz with resolution 20 Hz). It shows significant changes in the position of resonant frequency and amplitude of resonant peaks for different diameters of discs and under varying mechanical load conditions, implying variations in the mechanical boundary conditions on the structure. The highlight of our work is the proposed electrical equivalent circuit model for varying mechanically loaded conditions with the help of impedance technique. Our approach is simple and reliable, such that it is suitable for any structure whose accurate material properties and dimensions are unknown.

## 1. Introduction

### 1.1. Piezoelectric Materials

Piezoelectric effect is a phenomenon where mechanical deformation generates charge on its surface (direct effect) or electric field deforms the material (indirect effect). It is exhibited by quartz, semicrystalline polyvinylidene polymer, poly-crystalline piezoceramic, and many more [1,2]. Most commercial piezoelectric structures are made of perovskite lead zirconate titanate (PZT) due to its low cost, direct coupling, high voltage, and moisture tolerance. Due to concerns of lead toxicity, research on lead-free piezoelectric ceramics is gaining significant attention. Apart from the ceramics, flexible polymer based piezoelectric structures such as poly-(vinylidene flouride) (PVDF), and fiber-based structures are developed. The choice of a material depends on its properties, cost, operating frequency, temperature, and area of implementation. For any chosen material, a variety of piezoelectric structures are possible according to its design geometry or architecture [3,4].

### 1.2. Related Work

A piezoelectric element is a favorite choice as an energy harvester, sensor, or actuator implemented in medical instruments, wearable devices, automation, aviation, buildings, bridges, etc. For example, its electrical impedance, which is related to structural impedance, is used to monitor and detect mechanical cracks in most engineering structures [5,6]. Furthermore, it is suitable as both a wearable or nonwearable sensor to analyze human gait quality for patients with Parkinson’s disease or thrombosis, stroke, or diabetes, and also during rehabilitation and sports training [7,8,9,10,11,12,13]. Apart from sensing applications, they are also used for energy harvesting applications. Over the last few years, with growing interest in renewable and sustainable energy sources, piezoelectric elements gained attention as potential and reliable options to take advantage of ambient mechanical vibrations to generate usable electrical energy. They are used to operate sensor networks in remote areas by making them self-powering or to lower the dependency on batteries [14,15]. With every physical activity, there is mechanical vibration in human motion or vehicle movement, which piezoelectric elements can take advantage of to operate low power and miniaturized biomedical and portable devices for health monitoring of an individual [16,17].

For any piezoelectric energy harvesting system, there is a need of interface circuitry to serve the purpose [18,19,20,21,22]. The actual implementation of such integrated system is only possible after estimating it in simulation software. For such a scenario, the electrical impedance of its structure is a determining factor to optimize the electrical power harvest. With several application possibilities in broad range of operating frequency, varying temperature of experimental set-up, and varying load power demand of proposed application, the harvested power depends on the impedance matching between the load and input. The harvested power could be maximized with optimal impedance matching, without compelling the transducer to operate at resonant frequency. Moreover, by basic principle of piezoelectric element, its electrical power depends on the mechanical boundary conditions, which in turn is related to the impact of mechanical load on electrical impedance. Thus, apart from the importance of impedance matching for electrical power harvest, the impedance evaluation of our study allows us to cover the mechanical effects inside the circuit simulator, deriving several elements for equivalent circuit model [23,24,25].

For a system level application, and with several possible configurations and choices of materials, as discussed above, the modeling of piezoelectric element is a prerequisite to predict its performance and optimize its design. There are many modeling methods such as spring model, thermal analogy, and finite element to name a few, which are mostly based on mathematical equations of physical laws [24,26,27]. Since our focus is electrical simulation, we limit our discussion only to equivalent circuit models derived from electrical impedance.

## 2. Background

### 2.1. Existing Electrical Equivalent Models for Piezoelectric Elements

**Figure 1:**
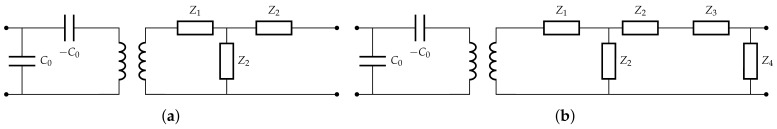
Mason’s model: (**a**) unloaded case and (**b**) loaded case.

There are seven main existing electrical equivalent models for piezoelectric elements: Mason, Van Dyke, Sherrit, Park, Guan, Easy, and Banerjee models, which will be discussed in this section. Mason’s equivalent model is one of the oldest electromechanical equivalent circuit with mutual transformations of mechanical and electrical parameters on both sides as shown in Figure 1. It is designed for both unloaded and loaded conditions, with impedance elements and electromechanical coupling factor dependent on several material constants. The material constants are real coefficients for lossless materials and complex coefficients for lossy materials. With addition of load to the element, the number of impedance elements increases on the mechanical port and derivation of such elements for lossy materials become more complicated. Another major drawback of Mason’s equivalent model is the negative capacitance on the electrical port, which is considered unrealistic [28,29,30,31].

Finite element analysis is one of the common techniques where mechanical parameters are used to derive their electrical analogies, from which the Van Dyke model is designed with lumped parameters. For the unloaded Van Dyke model, $L_{i}$, $C_{i}$ and $R_{i}$ are dependent on the mechanical equivalents as

(1) $$L_{i}=\frac{M}{\alpha^{2}},C_{i}=\frac{\alpha^{2}}{K_{m}},R_{i}=\frac{\eta }{\alpha^{2}}$$

where *M* is the rigid mass, $K_{m}$ is static stiffness of the PZT disc, α is force-voltage coupling factor, and η is its mechanical loss factor or equivalent damping.

**Figure 2:**
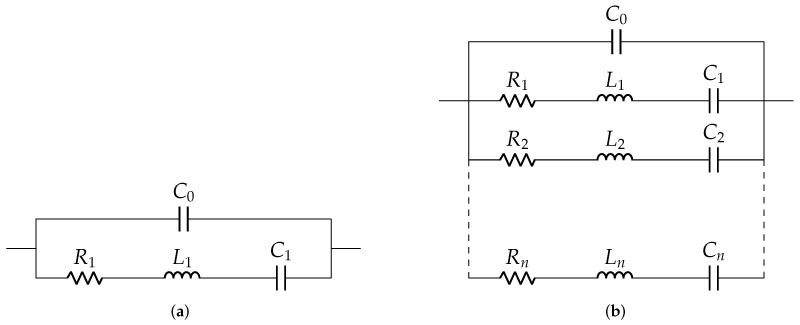
Van Dyke model: (**a**) unloaded case and (**b**) loaded case.

The Van Dyke model for unloaded and loaded conditions are shown in Figure 2. The number of parallel $R_{i}$-$C_{i}$-$L_{i}$ branches increases for loaded condition as the number of resonant peaks increases. The accuracy of such models depends on the dimension of the structures and material properties [27,32,33]. The Van Dyke electrical equivalent model perfectly fits only in the resonant ranges but not in the nonresonant ranges.

**Figure 3:**
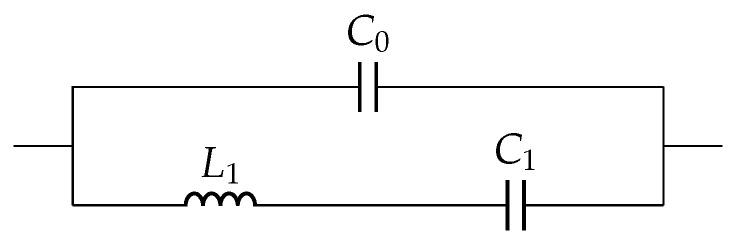
Sherrit model: unloaded case.

Due to inaccuracy in nonresonant ranges, which is more commonly found in materials with significant losses, it was improved in the Sherrit model using complex circuit components, which were calculated from the material constants in the mathematical equations [34]. The Sherrit model was only proposed for unloaded case as shown in Figure 3, with a pair of $L_{i}$-$C_{i}$ in parallel to the static capacitance $C_{0}$.

**Figure 4:**
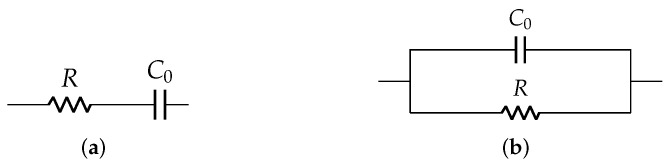
Park model: (**a**) series and (**b**) parallel.

However, in cases when the exact material composition is unknown, it is difficult to evaluate the material constants that govern such equations. For such cases, impedance response measurement provides a simple and straightforward modeling technique to derive electrical equivalent model. It is independent of structure geometry and material constants, and is more suitable for transducer application. The Park model was the basic circuit model with *R*-$C_{0}$ combination to demonstrate the equivalent circuit model, as shown in Figure 4. But this model did not consider the resonant peaks and was suitable only for lower frequency ranges [35].

**Figure 5:**
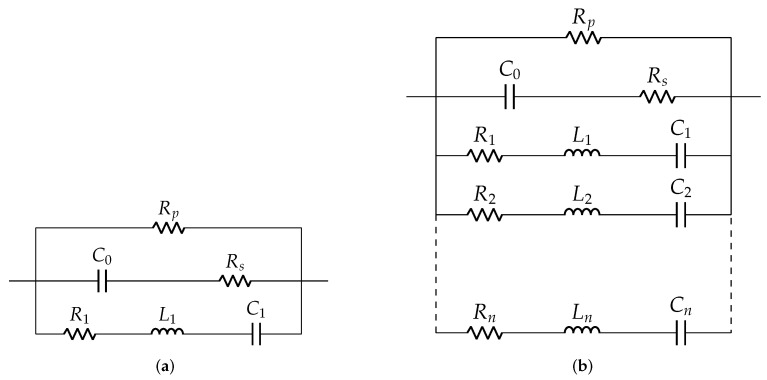
Guan model: (**a**) unloaded case and (**b**) loaded case.

The Guan models for unloaded and loaded cases are shown in Figure 5. It was the first model to consider resonant peaks, based on visual inspection of impedance response. It was inspired by the Van Dyke model and comes with additional components $R_{p}$ and $R_{s}$ to represent energy dissipation. The choice of values for $R_{p}$ and $R_{s}$ introduced inconsistency and inaccuracy in the fitted model, as energy dissipation was mainly dependent on amplitude and frequency of the excitation signal [36].

**Figure 6:**
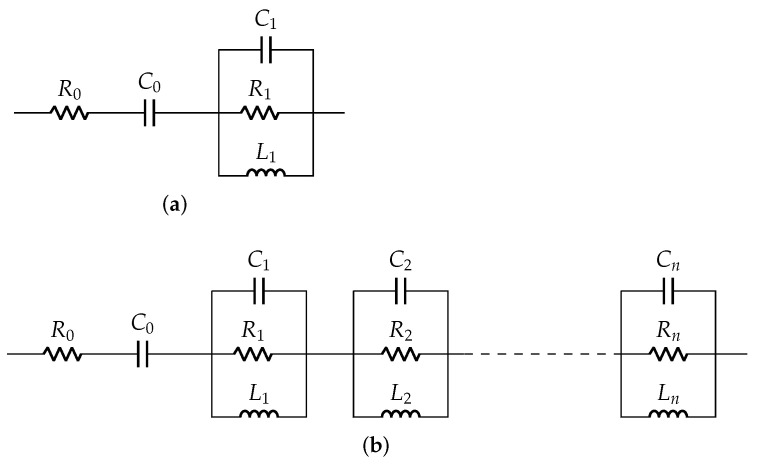
Easy model: (**a**) unloaded case and (**b**) loaded case.

**Figure 7:**
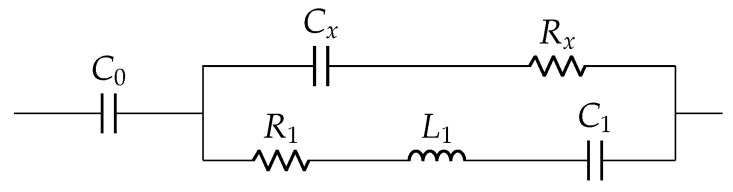
Banerjee model: unloaded case.

Based on impedance fitting, an $R_{0}$-$C_{0}$ circuit model with additional lumped parameters is derived to obtain an accurate equivalent circuit model for both mechanically loaded and unloaded conditions, separating the nonresonant and resonant parameters. The Easy model is a modified Van Dyke circuit with parallel $R_{i}$-$L_{i}$-$C_{i}$ with $C_{0}$ in series, as shown in Figure 6 [37], but based on impedance response. The Banerjee model is the most recent equivalent circuit model for unloaded case, where the concept of residual impedance was introduced for simplified modeling [25]. It estimated complex permittivity of the element by using the derived electrical model parameters and its dimensional constants. Its circuit schematic is shown in Figure 7.

### 2.2. Fundamental Equations

In support of the claims discussed in the above section, it is important for us to understand the governing equations of piezoelectric discs that relate the electrical and mechanical physical quantities. The constitutive equations of piezoelectric effect are:

(2) $$S=s^{E}T+d^{t}E$$

(3) $$D=dT+\epsilon^{T}E$$

where *S* is mechanical strain, *T* is mechanical stress, *E* is electric field, *D* is charge density, $s^{E}$ is stiffness at constant electric field *E*, *d* is matrix for the direct and reverse piezoelectric effect, and $\epsilon^{T}$ is dielectric permittivity under constant stress *T* [38].

The authors in [39] clearly summarized the electro-mechanical equations of an active material. For a piezoelectric disc of width *w*, thickness *h*, and length *l*, the mechanical impedance $Z_{mech}$, also known as short-circuit mechanical impedance, is defined as the ratio of excitation force *F* and velocity response *v*. It is written as:

(4) $$Z_{mech}=\frac{F}{v}=-\frac{K_{m}\left(1+\eta \right)}{\omega }\frac{k_{m}l}{\tan(k_{m}l)}$$

(5) ω=2πf

where ω is angular frequency and $k_{m}$ is the wave number.

Again, the electrical current *I* flowing through the disc is related to electric field *E* as follows:

(6) $$I=\omega Ewl\;\left[\;d^{2}Y\frac{Z_{mech}}{Z_{mech}+Z_{h}}\frac{\tan(k_{m}l)}{k_{m}l}+\epsilon^{T}-d^{2}Y^{E}\;\right]$$

where *Y* is the elastic modulus of the disc, and $Z_{h}$ is the impedance of host structure.

The applied electrical voltage *V* can be rewritten as:

(7) V=Eh

The inverse of electrical impedance is denoted by $Y_{elec}$ and is expressed as:

(8) $$Y_{elec}=\frac{I}{V}$$

Substituting from Equations (6) and (7) on (8), we get:

(9) $$Y_{elec}=\omega \frac{wl}{h}\;\left[\;d^{2}Y^{E}\frac{Z_{mech}}{Z_{mech}+Z_{h}}\frac{\tan(k_{m}l)}{k_{m}l}+\epsilon^{T}-d^{2}Y^{E}\;\right]$$

Rewriting Equation (9) with *V*, *I*, *P*, *A* and *v*, we get:

(10) $$\frac{I}{V}=Y_{elec}=\omega \frac{wl}{h}\;\left[\;d^{2}Y^{E}\frac{\frac{P\times A}{v}}{\frac{P\times A}{v}+Z_{h}}\frac{\tan(k_{m}l)}{k_{m}l}+\epsilon^{T}-d^{2}Y^{E}\;\right]$$

where

(11) $$P=\frac{F}{A}$$

and A is the cross sectional area of the disc.

Thus, the Equations (9) and (10) establish the relationship between mechanical and electrical impedance for any PZT disc [39]. As discussed in Section 1.2 and observed in the above equations, material properties and dimensions are crucial for determining the electro-mechanical impedance. But for commercial piezo structures where the accurate values of *w*, *l*, *h*, *d*, $Y_{E}$, $k_{m}$, η and *d* are unknown, the impedance analyzer provides a reliable and fast scope to understand the behavior of PZT discs and derive its electrical equivalent circuit.

## 3. Research Methodology

### 3.1. Proposed Work

Electrical impedance response measurement is a fundamental step to understand electrical behavior of sensors or energy harvesters. In our work, we make comparative study of electrical impedance response of piezoelectric elements for a wide range of frequencies 0.1–1000 kHz for mechanically unloaded and varying loaded cases. The existing equivalent models are derived for only few kHz of frequency range. The elements are not connected to any electrical load. Our focus is commercial piezoelectric elements because they have limited information about material properties to derive accurate model for system level application. In this paper, we use elements from Murata Electronics, which are quite popular due to their low-cost and ease of implementation. As discussed, due to limited information in the data-sheet, we use electrical impedance behavior as basis to derive equivalent circuit model for mentioned elements.

The purpose of our comparative study is to observe: (1) the influence of diameter on impedance response for mechanically unloaded piezoelectric discs of different diameters; and (2) the influence of varying mechanical load on the discs of specific diameter, which was not previously investigated for commercial piezoelectric elements using a wide range of frequencies. The change in electrical impedance response, observed in terms of resonant frequency shift and shape of resonant peaks (Q-factor), determine the values of electrical parameters in the derived model, which is crucial for accurate prediction of electrical behavior. Based on this study for a wide range of frequency, a new electrical equivalent circuit model for mechanically loaded condition is proposed for the first time. Considering the summary of all the existing models (from Section 2.1) mentioned in Table 1, we picked up the simplified technique from Easy modeling and residual impedance from Banerjee modeling to propose a new model to study the dependence of mechanical load on impedance response for a wide range of frequencies. The need for wide frequencies is important due to the harmonics present within nonlinear circuits, such as rectifiers containing diodes and switches, and thus offering nonlinearity in the system. The main novelty of this work is the measurement method to understand the influence of mechanical clamp on the impedance behavior of the piezoelectric elements.

### 3.2. Experimental Details

#### 3.2.1. Piezoelectric Discs

**Figure 8:**
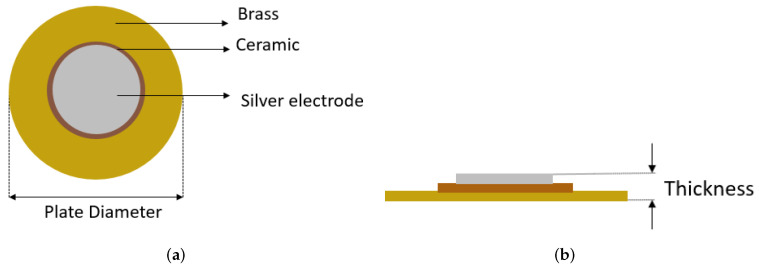
Piezoceramic disc: (**a**) top view and (**b**) side view.

The commonly used piezoelectric structure is a brass disc with a thin layer of PZT on its top (as shown in Figure 8) [40,41,42]. The brass disc, above which the PZT layer is deposited, is the host structure. Three different sizes of PZT discs were used in our work and their physical parameters are mentioned in Table 2.

#### 3.2.2. Test Equipment

The impedance response is measured by electrical impedance analyzer Keysight HP4194A, with the excitation voltage of −500 mV to +500 mV. This voltage induces mechanical expansion and contraction on the structure by indirect piezoelectric effect. The mechanical vibration as a result generates electrical response, which is measured in the form of current to calculate the electrical impedance.

### 3.3. Method for Electrical Impedance Measurement

**Figure 9:**
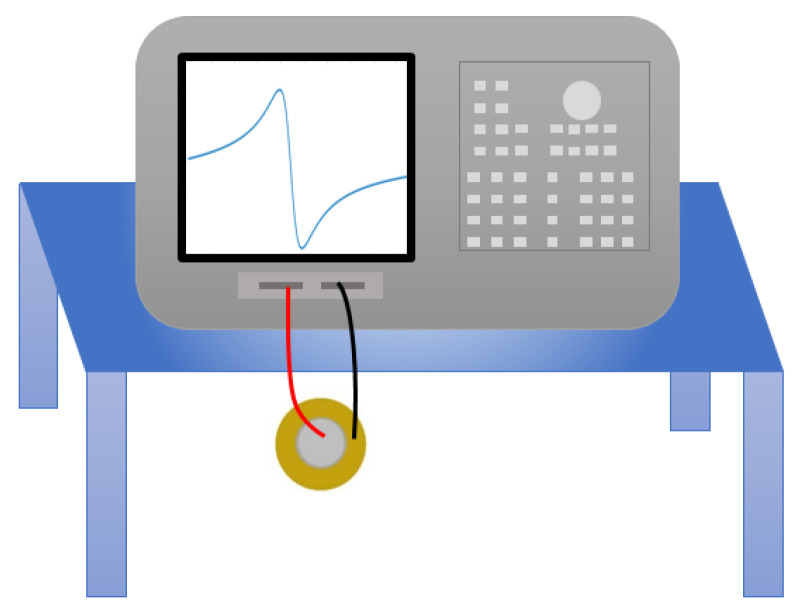
Experimental arrangement for mechanical unload.

**Figure 10:**
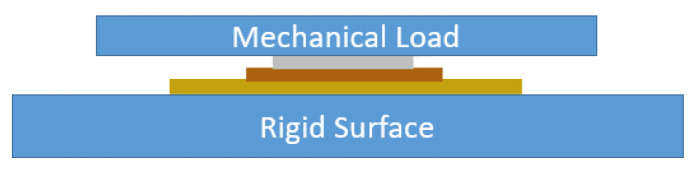
Experimental arrangement for mechanical load.

The measurement is performed for two conditions:

1. Without mechanical load: To understand the influence of plate diameters, three PZT discs with diameters of 12 mm, 20 mm and 27 mm are selected for measurement in the frequency range 0.1–1000 kHz with resolution of 20 Hz. PZT discs are suspended in the air as shown in Figure 9, such that they can vibrate freely to obtain the impedance response.
2. With mechanical load: To observe the influence of varying mechanical load, the impedance response of three discs are measured over the frequency range 0.1–1000 kHz with a resolution of 20 Hz at different load weights. The disc is placed flat on the table and then on top of it, equal loads are added one after another. Each load has a mass of 1.25 kg, which corresponds to force *F* = 12.25 N. Therefore, the pressure *P* equivalent to each mechanical load is derived from Equation (11). The definition of loading for the existing models mean that the piezoelectric element is mounted on a host structure, which we describe as mechanically unloaded in this paper. But in our case of mechanical loading, we apply a known mechanical load on top of the element as shown in Figure 10 below. The mechanical load on the arrangement generates normal displacement on the disc, perpendicular to its plane.

### 3.4. Derivation of Electrical Model Parameters

**Figure 11:**
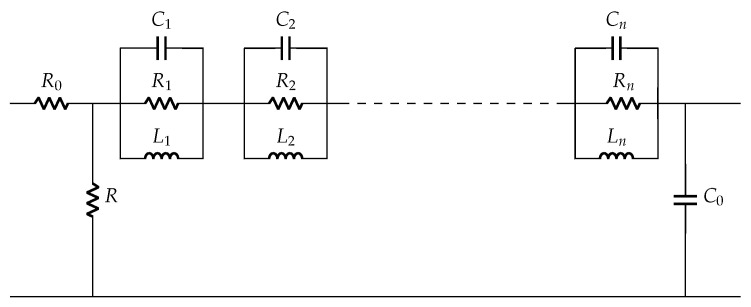
Equivalent circuit model of piezoelectric disc.

A pair of resistor *R* and capacitor $C_{0}$ is commonly used to model a PZT disc as shown in Figure 4, where *R* is a high value resistance and $C_{0}$ is a static capacitance. It is sufficient to understand the electrical behavior of the disc in low frequency applications. This *R*-$C_{0}$ model is extended with other elements ($R_{i}$-$C_{i}$-$L_{i}$) as shown in Figure 11 to understand its behavior for wide frequency range. The number of $R_{i}$-$C_{i}$-$L_{i}$ loop depends on the number of resonance peaks.

The $C_{0}$ is calculated from the measured impedance response $Z_{meas}$ at starting frequency 0.1 kHz, given by:

(12) $$Z_{C0}=Z_{meas(f=0.1\mathrm{kHz})}\sin\left(2\pi f\right)$$

(13) $$Z_{C0}=\frac{1}{2\pi fC_{0}}$$

The static capacitance $C_{0}$ is mainly dependent on the physical parameters of the disc with area *A* and plate separation *d*, and its dielectric constant ϵ which is given by:

(14) $$C_{0}=\frac{A\epsilon }{d}$$

The base resistance $R_{0}$ is determined from impedance at the last measured frequency, i.e., 1000 kHz:

(15) $$Z_{R0}=Z_{meas(f=1000\mathrm{kHz})}\cos\left(2\pi f\right)$$

As mentioned in the Banerjee model [25], we calculate the residual impedance $Z_{res}$ to identify the resonant peaks accurately as:

(16) $$Z_{res}=Z_{meas}-Z_{C0}$$

For each resonant frequency, $f_{i}$, $R_{i}$, $L_{i}$ and $C_{i}$ values are determined. $Q_{i}$ is the quality factor, which is related to the sharpness of each peak. $R_{i}$ is determined from the resistance at each peak, and the $L_{i}$ and $C_{i}$ values are calculated from following equations:

(17) $$\omega_{i}=2\pi f_{i}$$

(18) $$Q_{i}=\frac{\omega_{i}}{\mathrm{BW}}$$

where BW is the 3 dB bandwidth of the resonant peak

(19) $$L_{i}=\frac{R_{i}}{\omega_{i}Q_{i}}$$

(20) $$C_{i}=\frac{1}{{\omega_{i}}^{2}L_{i}}$$

The total impedance of a disc for unloaded case is given by:

(21) $$Z_{model}=\left(R_{0}||R+\sum_{n=1}^{N}Z_{Ri}\left|\right|Z_{Ci}\left|\right|Z_{Li}\right)\;\left|\right|\;\frac{1}{\omega C_{0}}$$

(22) $$Z_{model}=\left(R_{0}\left|\right|R+\sum_{n=1}^{N}Z_{i}\right)\;\left|\right|\;\frac{1}{\omega C_{0}}$$

(23) $$Z_{i}=\frac{1}{\frac{1}{R_{i}}+\omega C_{i}+\frac{1}{\omega L_{i}}}$$

where $Z_{i}$ is impedance due to a resonance peak.

The choice of best model fit depends on the difference between measured and simulated response (RE) and the correlation coefficient (COE) between them for the whole frequency range. The mathematical equations for RE and COE are:

(24) $$\mathrm{RE}=\frac{Z_{meas}-Z_{model}}{Z_{meas}}\times 100$$

(25) $$\mathrm{COE}=\frac{\sum (Z_{meas}-\bar{Z_{meas}})\left(Z_{model}-\bar{Z_{model}}\right)}{\sqrt{\sum (Z_{\mathrm{meas}}-\bar{Z_{meas}}{)}^{2}\sum {\left(Z_{model}-\bar{Z_{model}}\right)}^{2}}}$$

All calculations are performed in a Matlab program, and the corresponding LTspice model file is generated automatically. The Matlab program then calls LTspice to complete the simulation and process the data to generate the figures.

## 4. Results and Discussion

### 4.1. Without Mechanical Load

**Figure 12:**
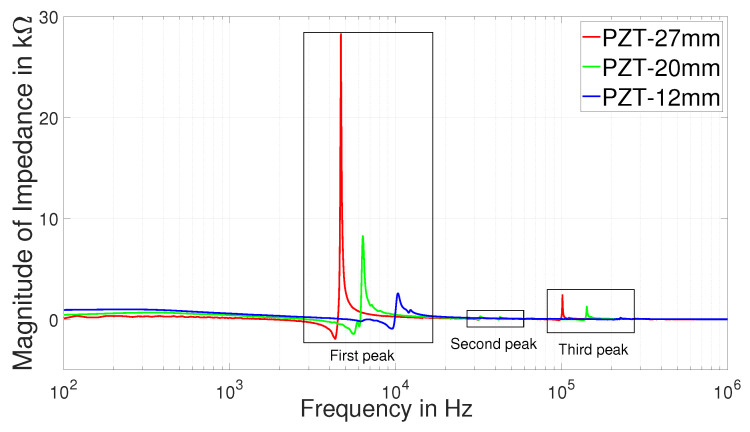
Magnitude of residual impedance of three PZTs.

**Figure 13:**
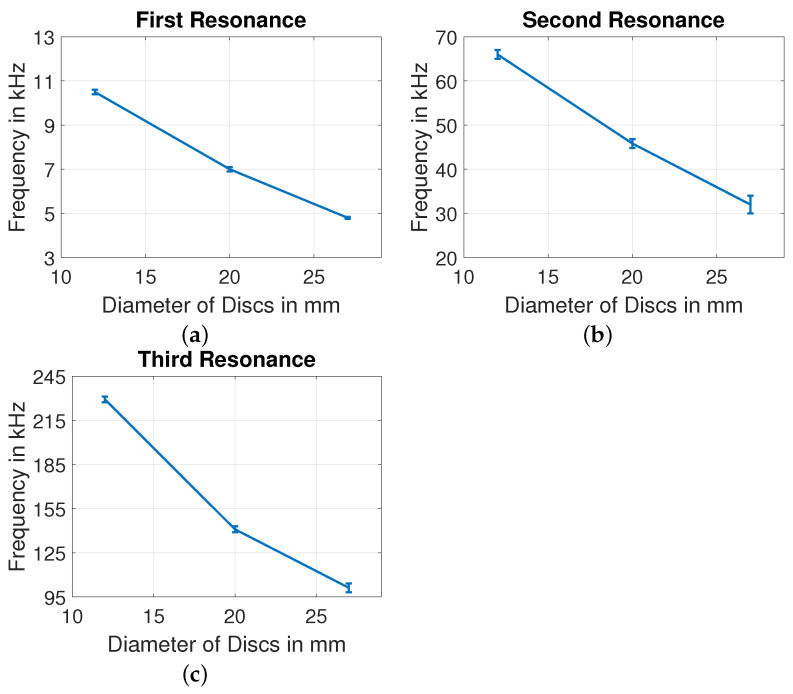
Influence of diameter at (**a**) first, (**b**) second, and (**c**) third resonance peaks.

The residual impedance $Z_{\mathrm{res}}$ in Equation (16) [25] of three PZT discs are shown in Figure 12, with rectangular blocks highlighted to show three resonant peaks. The peaks in this figure represent resonant peaks of each disc. With increase in plate diameter of the PZT discs, each resonant peak of a disc exhibits increasing impedance magnitude and increasing resonant frequency. For wide range of measured frequency, each PZT disc has three resonant peaks, where the second resonant peak has lowest amplitude. E.g., PZT-27 has three resonant peaks around 5, 33 and 100 kHz. The influence of diameter on three resonant frequencies of each disc are shown in Figure 13. The error bands considered in our figures are calculated from 5 sets of measurements for each type of disc.

**Figure 14:**
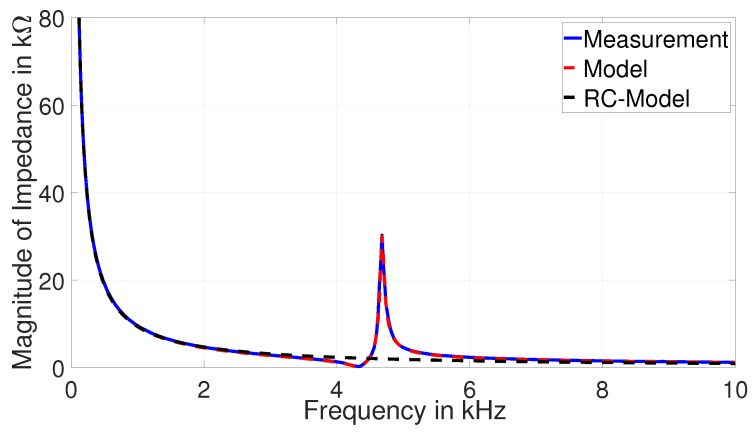
Comparison of magnitude of impedance of PZT-27.

**Figure 15:**
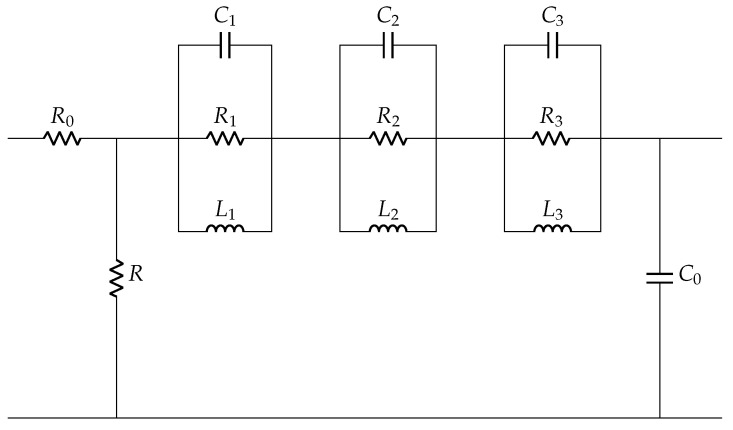
Equivalent circuit model of PZT-27 without mechanical load.

Based on the Equations (17)–(20) from [37], the values of model parameters are calculated to design an equivalent circuit model for mechanically unloaded condition. With Matlab-LTspice coupled simulations, the best fit of the first peak for PZT-27 is shown in Figure 14 from equivalent circuit model of the tested disc in Figure 15 and model parameters in Table 3. The decreasing impedance graph as ω→∞ in Figure 14 is associated with $Z_{C}0$, as given in Equation (13). Thus, $C_{0}$ is responsible for the capacitive behavior of the discs at low frequency range.

**Figure 16:**
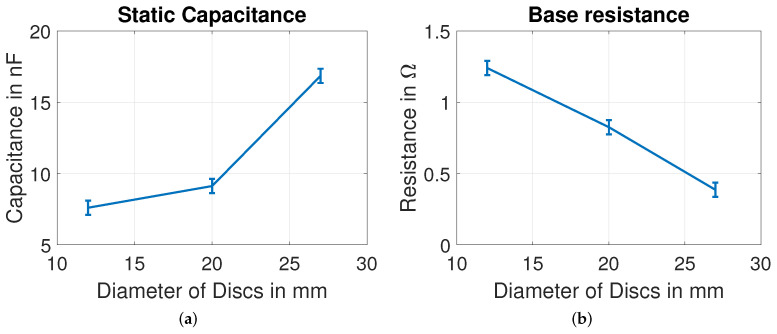
Influence of diameter on (**a**) static capacitance (**b**) base resistance of PZT discs.

**Figure 17:**
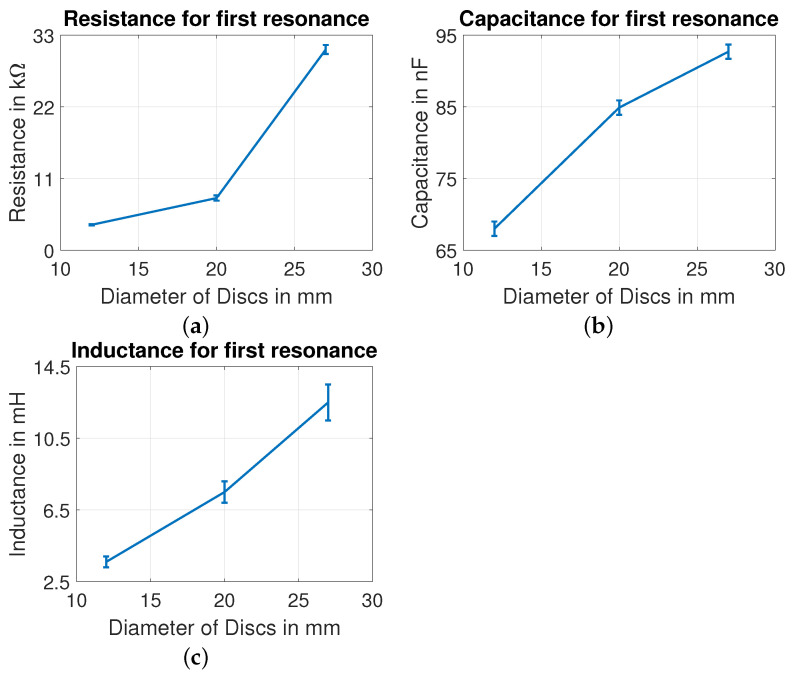
Influence of diameter on (**a**) resistance, (**b**) capacitance, and (**c**) inductance for first resonance.

In our measurements, it is observed that the electrical impedance response might vary slightly from one PZT disc of a particular size to another with different values of R${}_{i}$, L${}_{i}$ and C${}_{i}$, but the number of resonant peaks remains the same, with a fixed number of electrical parameters. Without mechanical load, the derived model parameters for PZT discs of different plate diameters are shown in Figure 16 and Figure 17. A gradual trend in $C_{0}$, $R_{0}$, $R_{1}$, $C_{1}$ and $L_{1}$ is observed with increase in plate diameter of PZT disc. As observed in Figure 16a, the static capacitance increases with increase in diameter of the disc, and it is in agreement with Equation (14). From the electrical–mechanical analogies in Equation (1), the increase in $R_{1}$, $C_{1}$ and $L_{1}$ with increase in diameter can be interpreted as increase in damping and decrease in stiffness of the discs.

### 4.2. With Mechanical Load

**Figure 18:**
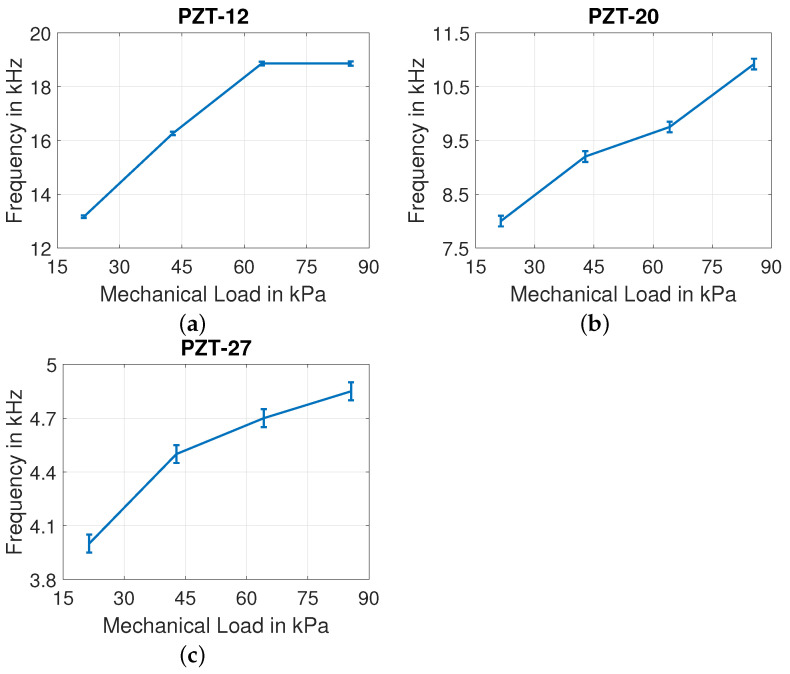
Influence of mechanical load on first resonant frequency of (**a**) PZT-12, (**b**) PZT-20, and (**c**) PZT-27.

**Figure 19:**
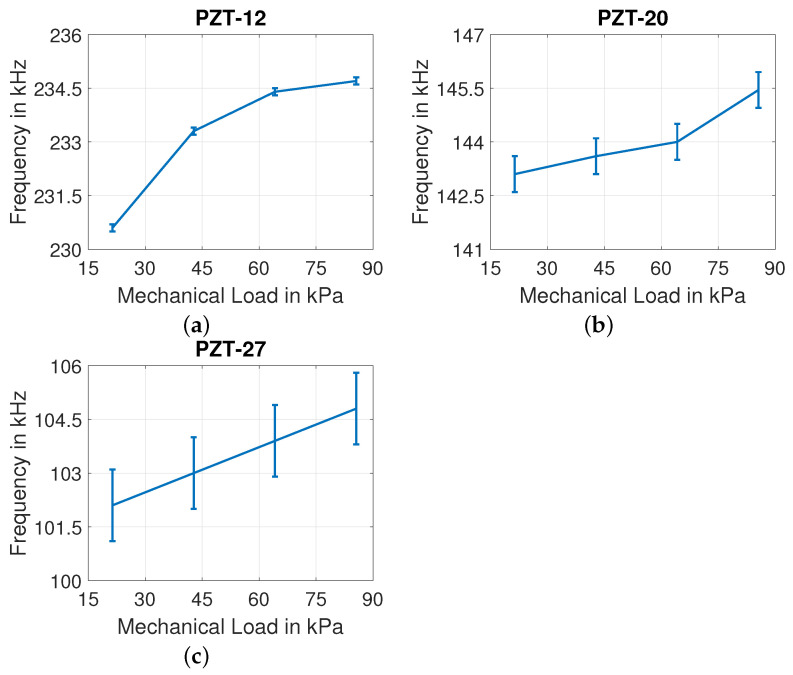
Influence of mechanical load on third resonant frequency of (**a**) PZT-12, (**b**) PZT-20, and (**c**) PZT-27.

For discs of all three diameters, the influence of mechanical load is observed in frequency shift of the first and third resonance peaks. The low quality factor of second resonance (as referred in Table 4) contributes to the disappearance of this peak due to mechanical load. So, the second resonant peaks of all discs vanish due to the influence of mechanical load on them. The impact of mechanical loading on frequency shift of PZT-12, PZT-20, and PZT-27 discs are shown in Figure 18 and Figure 19 for first and third resonant peaks.

**Figure 20:**
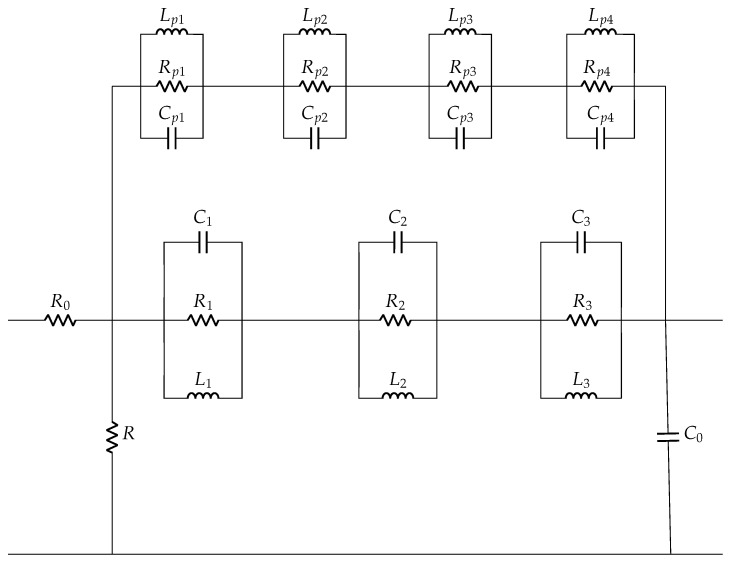
Equivalent circuit model of PZT-27 with mechanical load.

Based on the impedance data, a new equivalent circuit model derived for mechanically loaded case is shown in Figure 20. The equivalent circuit model without mechanical load (as shown in Figure 15) is extended with four sets of $R_{pi}$-$L_{pi}$-$C_{pi}$ in parallel to the three sets of resonance loops $R_{i}$-$L_{i}$-$C_{i}$. The influence of mechanical load in the optimized values of four sets of $R_{pi}$-$L_{pi}$-$C_{pi}$ for PZT-27 are noted in Table 4. For each addition of mechanical load 21.39 kPa, the values of $R_{pi}$-$L_{pi}$-$C_{pi}$ decrease, which is related to decrease in structural damping and increase in stiffness of the discs. Changes in electrical impedance response provide an understanding about changes in mechanical properties as a result of addition of mechanical load. What is interesting in our measurement is that loading only results in attenuation of resonant peaks instead of an increase in the number of resonant peaks, which was observed in other literature [37].

**Figure 21:**
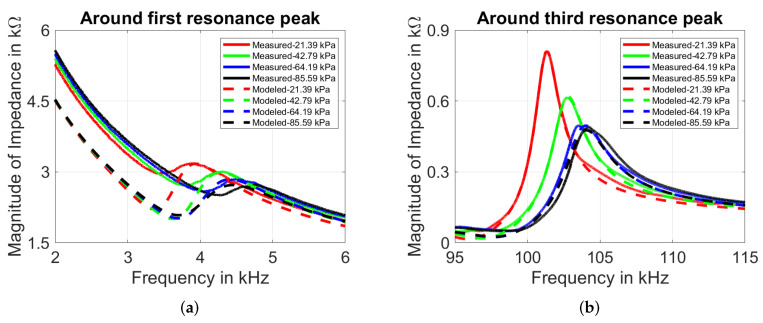
Influence of mechanical load on frequency dependence of PZT-27 disc around (**a**) first resonance and (**b**) third resonance peaks.

Figure 21a,b show measured and simulated impedance response of PZT-27. A rightward shift around first and third resonant frequencies with increase in mechanical load is observed for each case. The impact of mechanical load does not affect the quality factor of first resonant peaks. However, the peaks widen around the third resonance with increase in load, which is related to decrease in quality factor. The accuracy of simulated model to measured data can be interpreted from Table 5, which is acceptable considering the wide range of data.

## 5. Conclusions

Understanding of electrical and mechanical impedance response is fundamental for the application of piezoelectric elements. The occurrence of resonant peaks and nature of impedance magnitude depends on the structural and material specification of the discs. This paper provides insight into the electrical behavior of piezoelectric discs for a wide range of frequencies under mechanically loaded and unloaded conditions. Based on impedance equations, optimized electrical equivalent circuits of the discs were derived. The relative error for our modeling study is below 15%, and correlation coefficient is greater than 0.9985 for all cases considered in this work. The diameter of disc affects its model parameters, and so does the addition of mechanical load. The impedance magnitude resonance increases and resonant frequency decreases with an increase in the plate diameter. For mechanically loaded condition, the impedance magnitude at resonance decreases and resonant frequency increases with addition of load. This frequency shift provides hints of changes in the mechanical structures under various mechanical loading conditions. However, each disc has a unique impedance response, and the values of derived equivalent circuit parameters may vary slightly in their magnitudes. The technique used in this work can be implemented to understand impedance behavior and derive equivalent circuit model for other commercial piezoelectric discs as well.

With the existing single port models, our work attempts to derive a two-port model in future, where actual mechanical vibrations and their dynamic variations can be modeled inside the circuit simulator. Our study provides an overview of varying input mechanical effects on the piezoelectric elements. This will provide a reliable mechanical port in terms of electrical parameters to estimate correct prediction for energy efficiency of interface circuit and electro-mechanical power conversion for any device application.

## Figures and Tables

**Table 1:** Overview of existing models.

Name	Based on	Condition	Remarks
Mason	Material constants, field constants, and structure geometry	Both loaded and unloaded condition	Negative capacitance and determination of complex elements for lossy materials
Van Dyke	Material constants, field constants, and structure geometry	Both loaded and unloaded condition	Inaccurate in nonresonant frequency range and for lossy materials
Sherrit	Material constants, field constants, and structure geometry	Only unloaded condition	Determination of complex elements
Park	Impedance response	Only unloaded condition	Below ultrasonic frequency range
Guan	Impedance response	Both loaded and unloaded condition	Based on visual inspection, energy dissipation
Easy	Impedance response	Both loaded and unloaded condition	Limited frequency range, Simple
Banerjee	Impedance response	Only unloaded condition	Limited frequency range, Simple
Our model	Impedance response	Both loaded and unloaded condition	Wide frequency range, Simple

**Table 2:** Physical parameters of 3 perovskite lead zirconate titanate (PZT) discs.

Product Name	Plate Diameter (mm)	Structure Thickness (mm)	Ceramic Thickness (mm)	Mass (mg)
7BB-12-9	12	0.22	0.12	142
7BB-20-6	20	0.42	0.22	776
7BB-27-4	27	0.54	0.24	1968

**Table 3:** Model parameters for PZT-27 without mechanical load.

Resistance	Capacitance	Inductor	Quality Factor
*R* = e6 Ω			
$R_{0}$ = 0.4 Ω	$C_{0}$ = 16.85 nF		
$R_{1}$ = 30,300 Ω	$C_{1}$ = 95.40 nF	$L_{1}$ = 12.1 mH	$Q_{1}$ = 85
$R_{2}$ = 355 Ω	$C_{2}$ = 770.56 nF	$L_{2}$ = 32.1 μH	$Q_{2}$ = 55
$R_{3}$ = 2525 Ω	$C_{3}$ = 118.34 nF	$L_{3}$ = 20.9 μH	$Q_{3}$ = 190

**Table 4:** Model parameters for PZT-27 with mechanical load.

Load		Resistance		Capacitance		Inductor
No load		*R* = $10^{6}$ Ω				
		$R_{0}$ = 0.4 Ω		$C_{0}$ = 16.85 nF		
		$R_{1}$ = 30,300 Ω		$C_{1}$ = 95.40 nF		$L_{1}$ = 12.1 mH
		$R_{2}$ = 355 Ω		$C_{2}$ = 770.56 nF		$L_{2}$ = 32.1 μH
		$R_{3}$ = 2525 Ω		$C_{3}$ = 118.34 nF		$L_{3}$ = 20.9 μH
21.39 kPa		$R_{p1}$ = 900 Ω		$C_{p1}$ = 14.14 μF		$L_{p1}$ = 0.7162 H
		$R_{p2}$ = 1400 Ω		$C_{p2}$ = 105.2 nF		$L_{p2}$ = 3.3 H
		$R_{p3}$ = 1180 Ω		$C_{p3}$ = 10.5 nF		$L_{p3}$ = 230.1 μH
		$R_{p4}$ = 470 Ω		$C_{p4}$ = 101.5 nF		$L_{p4}$ = 11.08 H
42.79 kPa		$R_{p1}$ = 400 Ω		$C_{p1}$ = 0.795 μF		$L_{p1}$ = 0.0141 H
		$R_{p2}$ = 1300 Ω		$C_{p2}$ = 65.9 nF		$L_{p2}$ = 2.526 H
		$R_{p3}$ = 980 Ω		$C_{p3}$ = 9.7 nF		$L_{p3}$ = 190 μH
		$R_{p4}$ = 450 Ω		$C_{p4}$ 47.16 nF		$L_{p4}$ = 0.9549 H
64.19 kPa		$R_{p1}$ = 350 Ω		$C_{p1}$ = 0.641 μF		$L_{p1}$ = 0.0137 H
		$R_{p2}$ = 1000 Ω		$C_{p2}$ = 48.7 nF		$L_{p2}$ = 2.16 H
		$R_{p3}$ = 920 Ω		$C_{p3}$ = 8.5 nF		$L_{p3}$ = 181.58 μH
		$R_{p4}$ = 370 Ω		$C_{p4}$ = 40.4 nF		$L_{p4}$ = 0.8660 H
85.5 kPa		$R_{p1}$ = 300 Ω		$C_{p1}$ = 0.573 μF		$L_{p1}$ = 0.0129 H
		$R_{p2}$ = 850 Ω		$C_{p2}$ = 44.25 nF		$L_{p2}$ = 1.89 H
		$R_{p3}$ = 900 Ω		$C_{p3}$ = 8.47 nF		$L_{p3}$ = 181.51 μH
		$R_{p4}$ = 300 Ω		$C_{p4}$ = 40.4 nF		$L_{p4}$ = 0.5684 H

**Table 5:** Relative error and correlation coefficient for PZT-27.

Load	Relative Error	Correlation Coefficient
0	9.46 %	0.9985
21.39 (kPa)	11.76 %	0.9995
42.79 (kPa)	11.83 %	0.9995
64.19 (kPa)	12.76 %	0.9994
85.59 (kPa)	14.21 %	0.9995

## Data Availability

Not applicable.
